# Gut microbiome of Moroccan colorectal cancer patients

**DOI:** 10.1007/s00430-018-0542-5

**Published:** 2018-04-23

**Authors:** Imane Allali, Noureddine Boukhatem, Leila Bouguenouch, Hanaa Hardi, H. Abir Boudouaya, M. Belen Cadenas, Karim Ouldim, Saaïd Amzazi, M. Andrea Azcarate-Peril, Hassan Ghazal

**Affiliations:** 1 Laboratory of Biochemistry and Immunology, Faculty of Sciences, Mohammed V University in Rabat, Rabat, Morocco; 2 Laboratory of Physiology, Genetics and Ethnopharmacology, Faculty of Sciences of Oujda, University Mohammed Premier, Oujda, Morocco; 3 Polydisciplinary Faculty of Nador, University Mohammed Premier, Nador, Morocco; 4 Department of Molecular Genetics, University Hospital Hassan II of Fez, Fez, Morocco; 5 Department of Medicine, and Microbiome Core Facility, School of Medicine, University of North Carolina, Chapel Hill, NC USA; 6 National Center for Scientific and Technolgical Research, Rabat, Morocco

**Keywords:** Gut microbiome composition, Colorectal cancer, Bacterial community, 16S rRNA sequencing, Moroccan population

## Abstract

Although colorectal cancer is the third leading cause of death in Morocco, there are no studies of the microbiome changes associated with the disease in the Moroccan population. The aim of our study was to compare the stool microbiome of Moroccan cancer patients with healthy individuals. We analyzed the microbiome composition of samples from 11 CRC patients and 12 healthy individuals by 16S rRNA amplicon sequencing. Principal coordinate analysis of samples revealed defined cancer versus healthy clusters. Our findings showed that cancer samples had higher proportions of Firmicutes (*T* = 50.5%; *N* = 28.4%; *p* = 0.04), specifically of Clostridia (*T* = 48.3%; *N* = 19.0%; *p* = 0.002), and Fusobacteria (*T* = 0.1%; *N* = 0.0%; *p* = 0.02), especially of Fusobacteriia (*T* = 0.1%; *N* = 0.0%; *p* = 0.02), while Bacteroidetes were enriched in healthy samples (*T* = 35.1%; *N* = 62.8%; *p* = 0.06), particularly the class Bacteroidia (*T* = 35.1%; *N* = 62.6%; *p* = 0.06). *Porphyromonas, Clostridium, Ruminococcus, Selenomonas*, and *Fusobacterium* were significantly overrepresented in diseased patients, similarly to other studies. Predicted functional information showed that bacterial motility proteins, flagellar assembly, and fatty acid biosynthesis metabolism were significantly overrepresented in cancer patients, while amino acid metabolism and glycan biosynthesis were overrepresented in controls. This suggests that involvement of these functional metagenomes is similar and relevant in the carcinogenesis process, independent of the origin of the samples. Results from this study allowed identification of bacterial taxa relevant to the Moroccan population and encourages larger studies to facilitate population-directed therapeutic approaches.

**Electronic supplementary material:**

The online version of this article (10.1007/s00430-018-0542-5) contains supplementary material, which is available to authorized users.

## Introduction

Colorectal cancer (CRC) is one of the most common cancers worldwide, and is the third cause of cancer mortality in the world [1–3]. In Morocco, CRC is as prevalent, behind breast and cervical cancer for women, and lung and prostate cancer for men [4, 5]. The number of patients affected has increased over the last decade, with an increase in incidence from 6.0 per 100,000 to 10.4 per 100,000 from 2005 to 2008 in Rabat [5] and from 10.8 per 100,000 to 12.9 per 100,000 from 2004 to 2007 in Casablanca [4, 6].

CRC is a multifactorial disease with both environmental and genetic contributions to its pathogenesis. CRC can be classified by etiology as hereditary [7] (e.g. familial adenomatous polyposis due to an initiating mutation in the Adenomatous Polyposis Coli (APC) gene), inflammatory (associated with Crohn’s disease and ulcerative colitis), or sporadic (in more than 80% of cases) [8]. Risk factors for sporadic CRC include diet, age, alcohol consumption, smoking, physical activity and body mass index [9–12]. The incidence and mortality of CRC show geographical variation, with a high prevalence in Western countries, reflecting the importance of environmental factors [13–15]. Indeed, immigrants from low-incidence areas acquire similar CRC rates over time upon arrival in higher incidence areas, and eating habits likely contribute to this observation [16–19]. We have previously shown that geographic location and diet habits may impact the composition of the gut microbiome as reflected by significant differences in bacterial populations in tumor and tumor-adjacent tissues in individuals from Spain and the US [20]. Our study demonstrated an association between *Eikenella* and tumor tissues only in US individuals, while tumors from Spaniards were enriched for *Fusobacterium, Bulleida, Gemella, Parvimonas, Campylobacter*, and *Streptococcus*. In another study, a comparison of healthy African American and native African groups identified significant differences between the two populations due to higher dietary intakes of animal products by the African American population, with major butyrate-producing bacterial groups overrepresented in native African populations [16]. Finally, CRC rates in migrant groups from high-incidence southern European countries declined after more than 15 years of residence in Australia, approaching the rates of the host country [18].

Research studies indicate that composition and functionality of the gut microbiome play a major role in modulating CRC risk [12, 21–31]. Studies seeking to identify specific bacterial signatures associated with cancer incidence have not been successful [32–34] due to the complexity of the gut microbiome [35–38], and the diversity within and between individuals and populations [39, 40]. However, metagenomics studies have implicated certain bacterial species correlated with the presence of CRC [41–48]. Overrepresentation of species of *Fusobacterium* in CRC has been demonstrated in several studies in both stool and mucosal samples [20, 49, 50], raising the possibility that this species may play a causative role in carcinogenesis [51–53]. *Fusobacterium* is a rare inhabitant of the colon [54], but a well-known pathogen of the mouth, responsible for periodontitis and inflammation [55, 56]. A study by Rubinstein et al. showed that binding of *Fusobacterium nucleatum* to a specific receptor of the epithelial cells activated the proliferation of human colon cancer cells [57].

The Moroccan diet, rich in fruits, vegetables, and olive oil, is consistent with a Mediterranean diet. Despite dietary similarities, different regions display specific habits depending on cultural influences, religion, and lifestyles [58–60]. A study comparing dietary habits of Mediterranean populations from Spain, Morocco and Palestine reported high carbohydrate intake and low protein intake in Palestine compared to Spain and Morocco, while the Moroccan population had the highest consumption of fruits, vegetables, dairy products, and fish [61]. Considering the impact of diet on the composition of the gut microbiome [62–64], research studies from different geographic regions with different dietary habits are essential to advance the collective knowledge and allow tailored and effective CRC treatments. The aim of our study was to compare the composition of the gut microbiome of Moroccan CRC patients versus healthy individuals. We performed 16S rRNA amplicon sequencing of stool samples to determine composition, followed by predictive functional analysis of data. The findings of this study provide new insights on the gut microbiome composition and on specific bacterial communities related to CRC in an understudied population.

## Materials and methods

### Ethics statement

This study was approved by the University Hospital Center Hassan II of Fez, Morocco. A written informed consent was obtained from all patients and healthy individuals.

### Samples metadata and dietary questionnaires

Fecal samples were obtained from 11 colorectal cancer patients and 12 healthy subjects. Inclusion selection of CRC patients for this study was based on the following criteria: no gastrointestinal disorders, no antibiotic use during the last 3 months and those who had been recently diagnosed and had not yet started treatment. Inclusion criteria for healthy individuals were: absence of gastrointestinal disorders and no antibiotic treatment during the last 3 months. CRC and healthy individuals were of similar ages, came from the same region, and had similar diets and lifestyle. Individuals from the Oriental region and from Casablanca are served by the same University Hospitals. Colorectal cancer patients and healthy subjects were given a food survey questionnaire and were requested to report their diet over the past 5 days before collecting their stool samples. The food survey reports all food consumed by an individual during the day. From this survey, we measured the frequency of consumption of fruits, vegetables and red meat (e.g. times per day, daily, weekly). Dietary data were divided into two groups: (1) high consumption of fruits and vegetables/low consumption of meat and (2) low consumption of fruits and vegetables/high consumption of meat. Daily consumption of fruits, vegetables, and meat was measured in grams for each subject (patients and healthy individuals). Individuals in group 1 consumed more than 250 g of fruits and vegetables and less than 50 g of meat per day. Conversely, individuals in group 2 consumed less than 250 g of fruits and vegetables and more than 50 g of meat per day.

In addition to the food survey, information related to age, sex, body mass index (BMI) and family history of CRC were collected from the subjects. BMI was measured for both groups [BMI is calculated from body mass (*M*) and height (*H*). BMI = *M*/(*H* × *H*), where *M* = body mass in kilograms and *H* = height in meters]. Dietary data were self-reported.

### Sample storage and DNA isolation

All stool samples were collected in sterile Eppendorf tubes and then frozen at − 80 °C until DNA extraction. DNA isolation was carried out using the QIAmp DNA Stool kit supplied by Qiagen (Hilden, Germany). The subsequent steps were performed as recommended by the manufacturer’s protocol with minor modifications. Briefly, 200 mg of stool samples was added to a tube containing 1.4 ml buffer ASL. Samples were homogenized using a Tissue Lyser (Qiagen) for 1 min at 25 Hz. 15 ml of proteinase K and 200 ml AL buffer were added to samples, vortexed and incubated at 70 °C for 10 min according to the manufacturer’s instruction. Then, 200 ml of 100% ethanol was added to the mixture and they were transferred into a column. Following this, 500 ml of buffers AW1 and AW2 was added to the column separately and the flow-through was discarded in each step. Finally, 200 ml of buffer AE was added to the column to elute the DNA.

### 16S rRNA amplicon sequencing

Sequencing of 16S rRNA amplicons was done at the UNC Microbiome Core Facility. DNA was amplified using primers targeting the V1–V2 region of the bacterial 16S rRNA gene [65, 66] and overhang adapter sequences appended to the primer pair for compatibility with Illumina index and sequencing adapters. Master mixes contained 12.5 ng of total DNA, 2× KAPA HiFi HotStart ReadyMix (KAPA Biosystems, Wilmington, MA, USA). The thermal profile for the amplification of each sample had an initial denaturing step at 95 °C for 3 min, followed by a cycling of denaturing of 95 °C for 30 s, annealing at 55 °C for 30 s and a 30 s extension at 72 °C (25 cycles), a 5 min extension at 72 °C and a final hold at 4 °C. Each 16S rRNA amplicon was purified using AMPure XP reagent (Beckman Coulter, Indianapolis, IN, USA). Each sample was then amplified using a limited cycle PCR program, adding Illumina sequencing adapters and dual-index barcodes [index 1(i7) and index 2(i5)] (Illumina, San Diego, CA, USA) to the amplicon target. For the second round of amplification, the thermal profile consisted of an initial denaturing step at 95 °C for 3 min, followed by a denaturing cycle of 95 °C for 30 s, annealing at 55 °C for 30 s and a 30 s extension at 72 °C (8 cycles), and 5 min extension at 72 °C. The final libraries were again purified using AMPure XP reagent (Beckman Coulter), quantified and normalized prior to pooling. The DNA library pool was then denatured with NaOH, diluted with hybridization buffer and heat denatured before loading on the MiSeq reagent cartridge (Illumina) and on the MiSeq instrument (Illumina). Automated cluster generation and paired-end sequencing with dual reads were performed according to the respective manufacturer’s instructions.

### Bioinformatics sequencing data analysis

The Quantitative Insights Into Microbial Ecology (QIIME v.1.8.0) software pipeline [67] was conducted for the bioinformatics analysis of our bacterial 16S sequencing data. The raw sequences were demultiplexed and filtered; all reads with a length above 200 bp and with a quality score above 25 were kept. The resulting reads were clustered into operational taxonomic units (OTU) at 97% similarity threshold using UCLUST [68] from QIIME. After OTU picking, chimeras and singletons were removed using Chimera Slayer [69, 70]. Then, the sequences were aligned in order to build a phylogenetic tree using Fast Tree 2.1.3 [71]. The species level assignment was determined from the QIIME output using a biom file from the OTU picking. Additionally, to measure alpha diversity using observed species (*S*) and phylogenetic diversity (PD) metrics a random selection of 9090 sequences from each sample was used. Beta diversity and principal coordinates analysis (PCoA) were also calculated within QIIME using weighted and unweighted Unifrac distances [72] between samples at a depth of 9090 sequences per sample to evaluate dissimilarities between the samples. PD corresponds to the sum of branches on the phylogenetic tree among taxa occurring in a sample [73] and S is the number of OTUs per sample.

### Functional metagenome prediction

To predict the functional metagenome profiles from 16S rRNA amplicon sequencing input data, we used the Phylogenetic Investigation of Communities by Reconstruction of Unobserved States (PICRUSt) [74] (version 1.0.0) algorithm. Closed Reference OTUs were picked using UCLUST [68] against the GreenGenes database in order to create the OTU table for input into PICRUSt. The OTUs table result was normalized by dividing each OTU by the predicted 16S rRNA gene abundance before predicting the functional metagenome based on the KEGG orthology groups (KOs). The resulting functional metagenome by PICRUSt was used in the HMP Unified Metabolic Analysis (HUMAnN) [75] pipeline (version 0.99) to determine the presence or absence and the abundance of microbial KEGG pathways in our 16S rRNA amplicon sequencing data.

### Statistical analyses

*T*-Tests were performed to evaluate significant (*p* < 0.05) differences in phylogenetic diversity (PD) and species richness (*S*) indexes between healthy individuals and colorectal cancer patients. Analysis of Similarities (ANOSIM) and Permutational Multivariate Analysis of Variance (PERMANOVA) analyses were used to evaluate similarities between the two groups. The non-parametric Steel–Dwass method, which performs multiple comparisons while controlling the overall experiment-wise error rate, was applied to microbiome data. Significant differences (*p* < 0.05) in relative abundances of bacterial taxa and in metabolic pathways and enzymes between cohorts were computed using JMP Genomics (SAS, JMP Genomics 10.0). The Steel–Dwass All Pairs test corrects for multiple comparisons.

## Results

Eleven stool samples were collected from patients who had been diagnosed with CRC between October 2013 and December 2013, but who had not yet received treatment. The samples were collected from patients at the University Hospital Hassan II of Fez, Morocco. Twelve stool samples from healthy individuals were collected from the Oriental region (Northeastern area of Morocco) and Casablanca. CRC patients and healthy subjects were asked to give a detailed food record for the 5 days before sample collection, and data were collected regarding geographic origin, age, weight, family history, and risk factors. Characteristics of patients and healthy individuals are summarized in (Table 1). No statistical differences were observed between the two groups in age, sex and BMI with (*p* = 0.5), (*p* = 0.9), (*p* = 0.1) respectively.

**Table 1:** Summary of samples characteristics

Cohort	Colorectal cancer	Healthy
Number of samples	*N* = 11	*N* = 12
Age range (mean ± median)	(52.8 ± 54)	(49.3 ± 46)
20–29	2	0
30–39	0	4
40–49	0	3
50–59	5	1
60–69	2	3
70–79	2	1
Sex		
Female	7	11
Male	4	1
Tumor location		
Right colon	1	–
Sigmoid	1	–
Rectum	9	–
Body Mass Index (BMI) (mean ± median)	(25.3 ± 23.8)	(28.3 ± 26.8)
Underweight	1	0
Normal weight	4	1
Overweight	2	6
Obesity	1	2
Daily or almost daily consumption of fruits and/or vegetables		
Yes	3	6
No	2	6
Frequency of consumption of red meat		
Weak	–	1
Moderate	3	4
High	2	4
Family history of cancer		
Yes	–	2
No	–	–

After 16S rRNA amplicon sequencing of DNA extracted from stool samples, a total of 1,633,421 sequences passed our quality filtering (length > 200 bp, quality scores > 25). The average quality score was 35.5 ± 4.1, the average number of reads per sample was 67,505 ± 28,344, and the average of sequences length distribution was 315.2 ± 19.3. Almost the entirety of sequences (98.8%) was assigned to a taxonomic group, while 1.2% of the reads were unassigned. A total of 5081 Operational Taxonomic Units (OTUs) were identified in the cohort after clustering sequences at a 97% similarity threshold.

### The CRC microbiome had a higher diversity than the non-CRC microbiome

**Fig. 1:**
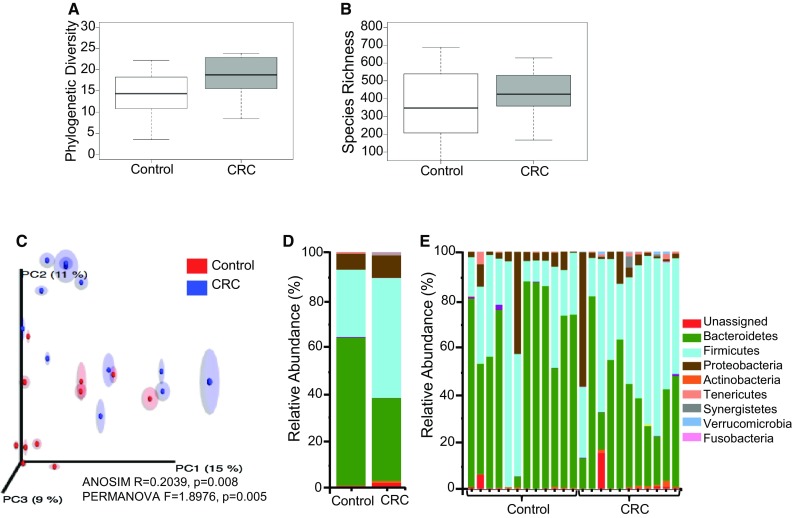
**a** Phylogenetic Diversity (PD) comparison between CRC and healthy individuals from the Moroccan population (*p < 0.1), **b** number of species identified in CRC and healthy individuals from the Moroccan population (*p > 0.1), **c** principal coordinates analysis—PCoA (unweighted UniFrac) of samples, **d** distribution of bacterial phyla in CRC versus healthy individuals, **e** distribution of bacterial phyla by individual (12 controls and 11 CRC)

Rarefaction analyses at a sampling depth of 9090 reads/sample were conducted to determine phylogenetic diversity (PD) and species richness (*S*) indexes (Fig. 1a, b). In contrast to our previous study on biopsy samples [20], we observed a trend towards CRC samples having higher PD and *S* values than healthy samples (*t* test *p* < 0.1).

Subsequent principal coordinates analysis (PCoA) with analysis of similarities (ANOSIM) and permutational multivariate analysis of variance (PERMANOVA) showed a low correlation between disease state in samples (ANOSIM, *R* = 0.2039, *p* = 0.008 and PERMANOVA, *F* = 1.8976, *p* = 0.005,) (Fig. 1c). No statistically significant differences were observed between control and CRC groups in the age category from 20 to 49 years old. However, a moderate but statistically significant effect was observed when we compared control and CRC groups in the second age category (ages 50–79) (ANOSIM, *R* = 0.3072, *p* = 0.036 and PERMANOVA, *F* = 1.7538, *p* = 0.036,). Comparison of samples according to sex, body mass index and diet showed no statistically significant differences between CRC and healthy groups.

### Diet associated with the microbiome composition in healthy and CRC subjects

**Fig. 2:**
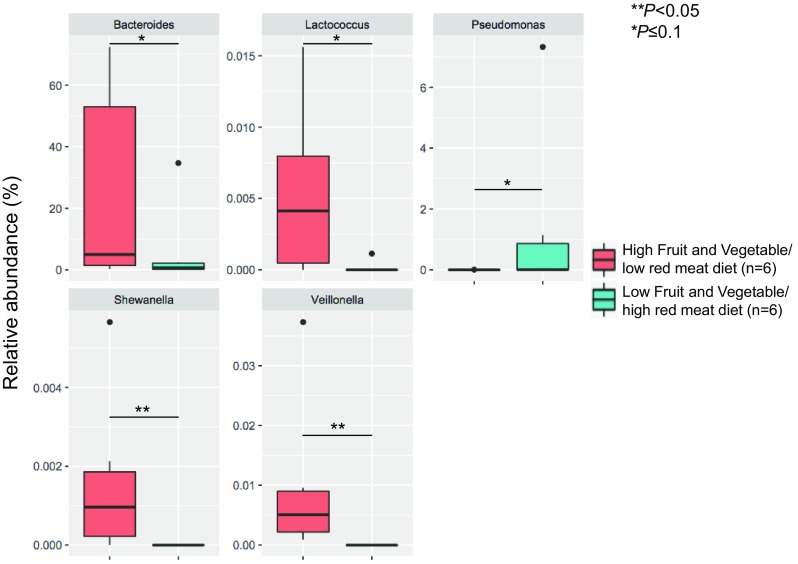
Relative abundances of significant bacterial genera in healthy individuals consuming a diet high in fruits and vegetables/low in red meat compared to healthy individuals following a diet low in fruits and vegetables/high in red meat (the boxplot scales are not the same)

To assess the impact of diet on the gut microbiome composition, we Evaluated the phylogenetic diversity (PD) and species richness (*S*) indexes in healthy individuals by comparing samples from individuals following a diet rich in fruits and vegetables and low in red meat consumption with samples from individuals with a diet low in fruits and vegetables/high in red meat. Although we were not able to conduct any statistical analysis due to the low number of samples (high fruits and vegetables/low red meat group *n* = 6; low fruits and vegetables/high red meat group *n* = 6), we observed that high fruits and vegetables/low red meat subjects had high values of phylogenetic diversity and species richness (PD = 14.3 ± 4.6, *S* = 367.0 ± 164.7 vs PD = 12.6 ± 5.2, *S* = 299.0 ± 177.4). *Veillonella, Shewanella, Lactococcus*, and *Bacteroides* were statistically overrepresented in the high fruits and vegetables/low red meat group, while *Pseudomonas* was statistically overrepresented in the low fruits and vegetables/high red meat group (*p* ≤ 0.1) (Fig. 2). We also evaluated the phylogenetic diversity (PD) and species richness (*S*) indexes in CRC patients. We found that patients having a diet rich in fruits and vegetables/low in red meat had slightly higher values of PD and S than patients having a diet low in fruits and vegetables/high in red meat (PD = 20.4 ± 0.8, *S* = 518.7 ± 99.2 vs PD = 20.0 ± 1.5, *S* = 445.5 ± 28.9). However, no significant differences in bacterial communities were observed in CRC patients according to type of diet.

### Gut microbiome composition of CRC and control stools

Our analysis showed that sequences clustered into 13 phyla, 26 classes, 48 orders, 92 families, and 165 genera. The most represented phyla in both CRC and controls were Bacteroidetes, Firmicutes, and Proteobacteria (Fig. 1d, e). Other phyla detected at low relative abundance (< 1.0%) were Actinobacteria, Cyanobacteria, Elusimicrobia, Fusobacteria, Lentisphaerae, Synergistetes, TM7, Tenericutes, and Verrucomicrobia. In the CRC group, Fusobacteria (CRC = 0.1% vs control = 0.0%), Firmicutes (CRC = 50.5% vs control = 28.4%) and Proteobacteria (CRC = 9.5% vs control = 6.8%) were overrepresented (Steel Dwass all pairs, *p* < 0.05), while Bacteroidetes (CRC = 35.1% vs control = 62.6%) were more prevalent in controls (*p* = 0.06). The predominant genera in both cohorts were *Bacteroides* and *Prevotella*. However, *Prevotella* was overrepresented in the control group while *Bacteroides* showed a non-significant overrepresentation in CRC (*p* ≤ 0.1). CRC stools were markedly different from controls, showing an overrepresentation of 33 genera (Table 2). The most significantly overrepresented species in normal samples compared to CRC samples were *Prevotella copri, Prevotella stercorea*, and *Faecalibacterium prausnitzii*, while for the CRC samples we found that *Collinsella aerofaciens* (Actinobacteria), [*Eubacterium*] *biforme* (Firmicutes), *Oxalobacter formigenes* (Proteobacteria), *Akkermansia municiphila* (Verrucomicrobia) and *Bacteroides fragilis* (Bacteroidetes) were significantly overrepresented.

**Table 2:** Comparison of bacterial genera significantly (Steel Dwass All Pairs, *p* values < 0.05) over- or underrepresented in CRC versus controls

Phyla	Genera	CRC	Control	*p* values
Overrepresented in CRC				
Actinobacteria	f_Coriobacteriaceae	0.002 ± 0.004	0.0003 ± 0.0005	0.0692
	*Atopobium*	0.003 ± 0.01	0.0003 ± 0.001	0.0612
	f_Coriobacteriaceae_Other	0.05 ± 0.08	0.006 ± 0.01	0.0681
Bacteroidetes	*Butyricimonas*	0.1 ± 0.4	0.06 ± 0.09	0.0333
	*Odoribacter*	0.3 ± 0.4	0.03 ± 0.05	0.0514
	*Parabacteroides*	1.4 ± 1.3	0.1363299 ± 0.1	0.021
	*Porphyromonas*	0.5 ± 0.9	0.0 ± 0.0	0.0047
	f_Rikenellaceae	2.6 ± 4.0	1.0 ± 1.2	0.0605
Firmicutes	f_[Mogibacteriaceae]	0.07 ± 0.06	0.009 ± 0.02	0.0199
	f_Christensenellaceae	8.4 ± 11.9	0.07 ± 0.1	0.0042
	*Christensenella*	0.002 ± 0.002	0.0 ± 0.0	0.0119
	f_Clostridiaceae	0.2 ± 0.2	0.06 ± 0.07	0.0247
	*Clostridium*	0.2 ± 0.1	0.06 ± 0.06	0.0246
	f_Dehalobacteriaceae	0.007 ± 0.01	0.0 ± 0.0	0.0659
	*Dehalobacterium*	0.02 ± 0.01	0.0008 ± 0.001	0.0035
	f_Lachnospiraceae	3.1 ± 2.3	1.3 ± 1.6	0.0074
	*[Ruminococcus]*	0.3 ± 0.5	0.1 ± 0.2	0.0365
	f_Lachnospiraceae_Other	2.1 ± 1.9	1.8 ± 2.7	0.0525
	*Peptostreptococcus*	0.06 ± 0.1	0.0001 ± 0.0003	0.0157
	f_Ruminococcaceae	0.1 ± 0.07	0.03 ± 0.04	0.0023
	*Oscillospira*	1.3 ± 1.1	0.3 ± 0.3	0.0012
	*Ruminococcus*	0.6 ± 0.4	0.4 ± 0.6	0.021
	f_Ruminococcaceae_Other	1.3 ± 1.0	0.3 ± 0.5	0.0455
	*Selenomonas*	0.07 ± 0.3	0.0 ± 0.0	0.0119
	f_Erysipelotrichaceae	0.6 ± 1.5	0.06 ± 0.1	0.0514
	*[Eubacterium]*	0.6 ± 0.5	0.06 ± 0.1	0.0246
	*Holdemania*	0.01 ± 0.01	0.003 ± 0.005	0.073
Fusobacteria	*Fusobacterium*	0.08 ± 0.1	0.0003 ± 0.0009	0.0348
Proteobacteria	*Oxalobacter*	0.03 ± 0.02	0.01 ± 0.02	0.0093
	f_Desulfovibrionaceae	0.01 ± 0.01	0.004 ± 0.009	0.0341
	*Bilophila*	0.2 ± 0.2	0.04 ± 0.05	0.0066
Synergistetes	f_Synergistaceae_Other	0.04 ± 0.09	0.0 ± 0.0	0.0659
Verrucomicrobia	*Akkermansia*	0.4 ± 0.4	0.01 ± 0.03	0.0399
Overrepresented in controls				
Firmicutes	*Megamonas*	0.0 ± 0.0	0.3 ± 1.5	0.0453
	*Mitsuokella*	0.02 ± 0.08	0.7 ± 0.8	0.0529
Proteobacteria	f_Bradyrhizobiaceae	0.0 ± 0.0	0.001 ± 0.002	0.0399

Numbers represent relative abundance (%) ± standard deviation

### Predicted functional differences between the CRC and control cohorts

**Fig. 3:**
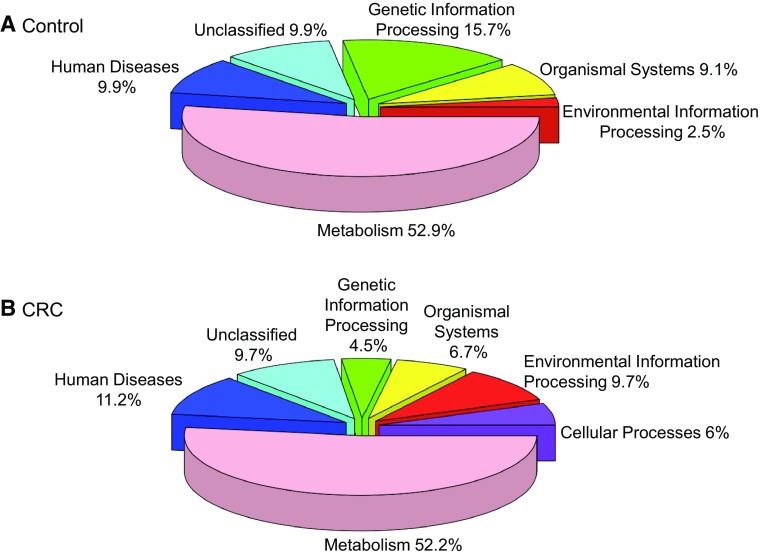
Relative abundance of the predicted functional pathways in control versus CRC individuals

We used the Phylogenetic Investigation of Communities by Reconstruction of Unobserved States (PICRUSt) [75] to identify differences in metagenome functional prediction based on Greengenes 16S rRNA database and KEGG Orthologs (KO). A total of 328 functional metagenomes were predicted in both CRC and control cohorts (Fig. 3, Supplementary Table 1). Cellular processes (bacterial chemotaxis, bacterial motility proteins, and flagellar assembly), environmental information processing (membrane transport and signal transduction), lipid (fatty acid biosynthesis and fatty acid metabolism) and carbohydrate metabolism (pentose phosphate pathway), and xenobiotics biodegradation and metabolism were overrepresented in the CRC cohort (Steel Dwass All Pairs, *p* < 0.05). In contrast, genetic information processing (chaperones and folding catalysts, RNA degradation, and protein processing in endoplasmic reticulum), organismal systems (carbohydrate digestion and absorption, protein digestion and absorption, and NOD-like receptor signaling pathway), amino acid metabolism (glycine, serine and threonine metabolism), energy metabolism (oxidative phosphorylation), glycan biosynthesis and metabolism (glycosyltransferases and lipopolysaccharide biosynthesis) and metabolism of other amino acids (glutathione metabolism) were significantly overrepresented in the control cohort. The significantly overrepresented enzymes between CRC and controls samples are listed in (Table 3).

**Table 3:** Enzymes significantly over- or underrepresented in the colorectal cancer and control samples (*p* < 0.05)

Enzymes	EC	*p* values	Control	CRC	Ratio	Fold change
Overrepresented in the Control Samples						
*O*-Succinylbenzoic acid–CoA ligase	EC:6.2.1.26	0.0127	0.6	3.2	0.2	−1.5
Acid phosphatase (class A)	EC:3.1.3.2	0.0178	0.7	4.3	0.1	− 1.7
Carbonic anhydrase	EC:4.2.1.1	0.021	0.6	3.1	0.2	− 1.5
Ribonucleoside-diphosphate reductase beta chain	EC:1.17.4.1	0.0247	0.6	3.0	0.2	− 1.5
Alpha-amylase	EC:3.2.1.1 2	0.0337	0.6	3.1	0.2	− 1.5
lysozyme	EC:3.2.1.17	0.0337	0.7	3.7	0.1	− 1.6
Carbonyl reductase (NADPH)	EC:1.1.1.184	0.0385	0.9	11.2	0.08	− 2.5
Dipeptidase E	EC:3.4.13.21	0.0392	0.6	2.7	0.2	− 1.4
Naphthoate synthase	EC:4.1.3.36	0.0392	0.6	3.1	0.2	− 1.5
Putative metalloprotease	EC:3.4.24.-	0.0392	0.7	3.6	0.1	− 1.6
Isochorismate synthase	EC:5.4.4.2	0.0455	0.6	3.2	0.2	− 1.5
Phosphatidylethanolamine N-methyltransferase	EC:2.1.1.17	0.0478	0.9	44.4	0.02	− 3.8
2-Hydroxy-3-oxopropionate reductase	EC:1.1.1.60	0.0289	0.4	1.7	0.2	− 1.4
Overrepresented in the Colorectal Cancer Samples						
2-Dehydro-3-deoxygalactonokinase	EC:2.7.1.58	0.0106	0.4	1.7	0.2	− 1.4
2-Phosphosulfolactate phosphatase	EC:3.1.3.71	0.0246	0.2	1.2	0.1	− 1.7
3-Dehydro-l-gulonate 2-dehydrogenase	EC:1.1.1.130	0.0247	0.4	1.7	0.2	− 1.4
3-Hydroxybutyryl-CoA dehydratase	EC:4.2.1.55	0.021	0.3	1.5	0.2	− 1.4
5-Methylthioadenosine/*S*-adenosylhomocysteine deaminase	EC:3.5.4.- 3.5.4.28	0.0455	0.3	1.5	0.2	− 1.4
Acetaldehyde dehydrogenase (acetylating)	EC:1.2.1.10	0.0335	0.5	2.1	0.2	− 1.3
Acetate CoA-transferase alpha subunit	EC:2.8.3.8	0.0247	0.4	1.9	0.2	− 1.3
Acetyl-CoA synthetase (ADP-forming)	EC:6.2.1.13	0.0384	0.8	5.3	0.1	− 1.8
Acetylglutamate kinase	EC:2.7.2.8	0.0337	0.4	1.6	0.2	− 1.4
Acyl-CoA dehydrogenase	EC:1.3.99.- 2	0.0023	0.1	1.1	0.08	− 2.4
Adenylate cyclase, class 2	EC:4.6.1.1	0.0221	0.3	1.5	0.2	− 1.4
Alanine-synthesizing transaminase	EC:2.6.1.-	0.0454	0.3	1.5	0.2	− 1.4
Aminotransferase	EC:2.6.1.- 2	0.0106	0.3	1.5	0.2	− 1.5
Arginine decarboxylase	EC:4.1.1.19 4	0.0023	0.2	1.3	0.1	− 1.6
Asparagine synthase (glutamine-hydrolysing)	EC:6.3.5.4	0.0392	0.3	1.6	0.2	− 1.4
Aspartate aminotransferase	EC:2.6.1.1 4	0.0151	0.3	1.5	0.2	− 1.4
Beta-glucosidase	EC:3.2.1.21 2	0.0455	0.4	1.7	0.2	− 1.4
Beta-phosphoglucomutase	EC:5.4.2.6	0.021	0.3	1.5	0.2	− 1.4
Butyryl-CoA dehydrogenase	EC:1.3.8.1	0.021	0.3	1.4	0.2	− 1.5
C4-Dicarboxylate-binding protein DctP		0.0035	0.2	1.3	0.1	− 1.6
Carbon monoxide dehydrogenase/acetyl-CoA synthase subunit alpha	EC:1.2.7.4 1.2.99.2 2.3.1.169	0.021	0.2	1.4	0.2	− 1.5
Carbon-monoxide dehydrogenase gamma subunit	EC:1.2.99.2	0.0337	0.3	1.4	0.2	− 1.5
Cell division inhibitor SepF		0.0455	0.3	1.5	0.2	− 1.4
Chemotaxis protein CheD	EC:3.5.1.44	0.0015	0.1	1.2	0.1	− 1.8
Chemotaxis protein methyltransferase CheR	EC:2.1.1.80	0.0074	0.2	1.3	0.1	− 1.6
Cyanophycinase	EC:3.4.15.6	0.0067	0.01	1.01	0.01	− 4.1
Cysteine desulfurase	EC:2.8.1.7	0.0247	0.3	1.5	0.2	− 1.4
Cystine transport system ATP-binding protein	EC:3.6.3.-	0.0337	0.3	1.5	0.2	− 1.4
D-proline reductase (dithiol) PrdE	EC:1.21.4.1	0.0422	0.3	1.5	0.2	− 1.4
Dihydroflavonol-4-reductase	EC:1.1.1.219	0.0074	0.2	1.3	0.1	− 1.7
Fatty acid synthase, bacteria type	EC:2.3.1.-	0.002	0.04	1.04	0.04	− 3.1
Flagellar assembly factor FliW		0.0015	0.1	1.2	0.1	− 1.9
Flagellar assembly protein FliH		0.0056	0.2	1.2	0.1	− 1.7
Flagellar biosynthesis protein		0.0062	0.2	1.3	0.1	− 1.6
Flagellar hook protein FlgE		0.0151	0.2	1.3	0.1	− 1.6
Flagellar protein FlaG		0.0074	0.2	1.2	0.1	− 1.7
Fructose-6-phosphate aldolase 2	EC:4.1.2.-	0.0089	0.3	1.5	0.2	− 1.4
Galactonate dehydratase	EC:4.2.1.6	0.0178	0.2	1.3	0.1	− 1.6
Glucoamylase	EC:3.2.1.3	0.0305	0.05	1.05	0.05	− 2.9
Glucose 1-dehydrogenase	EC:1.1.1.47	0.0046	0.2	1.2	0.1	− 1.7
Glutamate formiminotransferase	EC:2.1.2.5	0.0392	0.4	1.8	0.2	− 1.3
Glutamate synthase (ferredoxin)	EC:1.4.7.1	0.0178	0.3	1.5	0.2	− 1.4
Glutamine amidotransferase	EC:2.4.2.-	0.0392	0.3	1.6	0.2	− 1.4
Glycerol kinase	EC:2.7.1.30	0.0392	0.3	1.5	0.2	− 1.4
Glycine reductase	EC:1.21.4.2	0.0023	0.2	1.3	0.1	− 1.6
Histidinol-phosphatase (PHP family)	EC:3.1.3.15	0.0106	0.3	1.5	0.2	− 1.4
Inosose isomerase	EC:5.3.99.-	0.0392	0.3	1.4	0.2	− 1.5
l-Asparagine permease		0.0287	0.6	3.08	0.2	− 1.5
Lipopolysaccharide transport system permease protein		0.021	0.4	1.7	0.2	− 1.4
Mannonate dehydratase	EC:4.2.1.8	0.0455	0.4	1.8	0.2	− 1.3
Methyl-galactoside transport system substrate-binding protein		0.0151	0.3	1.5	0.2	− 1.4
Motility quorum-sensing regulator/GCU-specific mRNA interferase toxin		0.021	0.03	1.03	0.03	− 3.4
*N*-Acetylglucosamine-6-phosphate deacetylase	EC:3.5.1.25	0.0392	0.4	1.7	0.2	− 1.4
*N*-Glycosylase/DNA lyase	EC:3.2.2.- 4.2.99.18 2	0.0089	0.2	1.3	0.2	− 1.5
Ornithine carbamoyltransferase	EC:2.1.3.3	0.0289	0.3	1.5	0.2	− 1.4
Phosphatidylglycerol:prolipoprotein diacylglycerol transferase	EC:2.-.-.-	0.0289	0.3	1.5	0.2	− 1.4
Purine catabolism regulatory protein		0.0178	0.4	1.7	0.2	− 1.4
Putative glutamine amidotransferase		0.0074	0.2	1.4	0.2	− 1.5
Pyrimidine-specific ribonucleoside hydrolase	EC:3.2.-.-	0.0247	0.3	1.5	0.2	− 1.4
Pyruvate ferredoxin oxidoreductase, alpha subunit	EC:1.2.7.1	0.0089	0.2	1.2	0.1	− 1.7
Serine/threonine-protein kinase Stk1	EC:2.7.11.-	0.047	0.03	1.03	0.03	− 3.3
Sirohydrochlorin cobaltochelatase	EC:4.99.1.3 2	0.0283	0.04	1.05	0.04	− 3.07
Superoxide dismutase	EC:1.15.1.1	0.0162	0.1	1.2	0.1	− 1.8
Threonine 3-dehydrogenase	EC:1.1.1.103	0.0392	0.4	1.7	0.2	− 1.4
Two-component system, AgrA family, sensor histidine kinase AgrC	EC:2.7.13.-	0.0455	0.3	1.4	0.2	− 1.5
Two-component system, OmpR family, alkaline phosphatase synthesis response regulator PhoP		0.0178	0.3	1.5	0.2	− 1.4
UDP-*N*-acetyl-d-glucosamine dehydrogenase	EC:1.1.1.-	0.0138	0.1	1.1	0.09	− 2.3
Virulence factor		0.0289	0.2	1.3	0.1	− 1.6
Xanthine phosphoribosyltransferase	EC:2.4.2.22	0.0488	0.5	2.3	0.2	− 1.4

## Discussion

CRC incidence in African populations is low compared to European and North American populations. This low incidence has been attributed to anthropomorphic or environmental factors [76, 77]. However, incidence has been increasing over the last decade due to the westernization of the diet in many African countries [4–6, 13, 14, 78, 79].

Studies to understand the impact of geographical and cultural differences affecting potential roles of the gut microbiome on CRC, especially in understudied populations, are essential. In this analysis, we compared 11 stool samples from CRC patients with 12 stool samples from healthy Moroccan subjects. We observed a trend towards a higher phylogenetic diversity (PD) and species richness (*S*) in CRC versus controls, but the differences were not significant. Other studies showed no significant changes in diversity and species richness between CRC and healthy subjects [80–83] and similar observations were made in tissue samples. A comparison of 90 matched pairs of colorectal carcinoma and tumor-adjacent (normal) tissues from cohorts from the US and Spain showed no significant differences between normal and tumor tissues for both cohorts [20]. In contrast, other studies have reported significant differences in diversity and species richness in both tissue [84–86] and stool samples [21].

The genus *Bacteroides* was overrepresented in CRC while *Prevotella* was increased significantly in controls. Specifically, *Bacteroides fragilis* was more common in CRC patients. Our findings agree with previous studies that showed that *Bacteroides* were enriched in CRC patients [20, 34, 75]. *Prevotella* has been repeatedly associated with diets rich in fiber, while diets rich in fat and animal protein are conducive to a *Bacteroides*-dominated gut microbiota [62, 63, 87]. More recently, the enterotoxigenic *Bacteroides fragilis toxin* (ETBF) has been shown to cause chronic inflammation that could promote CRC [34, 87–90]. One of the three isoforms of ETBF indirectly induces cleavage of E-cadherin, resulting in increased epithelial cell permeability and exposure to bacterial antigens, which contribute to chronic inflammation [91–93]. ETBF has been associated with inflammatory bowel disease and CRC. It is characterized by the activation of Stat3 in mucosal immune and epithelial cells, with a subsequent colonic mucosal Th17 response that induces robust colonic tumors [94]. Additionally, it has been shown that treatment with antibody-mediated IL-17, a key cytokine amplifying Th17, reduced ETBF and tumor formation [95].

Similarly, *Fusobacterium* was overrepresented in our CRC cohort. This genus has been recurrently associated with CRC [20, 48, 50–53, 57, 96–98]. Moreover, *Fusobacterium* was not detected in healthy control samples, as it is a relatively uncommon bacterium in the gut microbiome. *F. nucleatum* [51] has been associated with CRC through its ability to stimulate the proliferation of tumor cells through the FadA (fluffy autolytic dominant A) adhesion gene [99–101]. FadA expression has been associated with increased expression of oncogenic and inflammatory genes; it may have a major role in the transformation of epithelial cells and promotion of colon tumorigenesis. Additionally, FadA binds E-cadherin, activating the beta-catenin signaling pathway, promoting the gut inflammatory response [57]. Administration of *F. nucleatum* to Apc(Min/+) mice increased the number of colon tumors and recruited tumor-infiltrating myeloid cells, inducing a pro-inflammatory state similar to that observed in humans [52].

Our study confirmed the role of other bacterial biomarkers in CRC, including *Porphyromonas*, overrepresented in CRC samples in accordance with previous reports [21, 83, 84, 102]. An association between oral bacteria, periodontal disease, and cancer has been established, specifically between *Porphyromonas gingivalis* and *F. nucleatum* [99, 103–106]. *Porphyromonas* has been associated with oral periodontal disease. It can penetrate periodontal tissue and alters the composition of the oral microbiome [89, 107]. *Porphyromonas gingivalis* has been linked to orodigestive cancer [108], pancreatic cancer [109] and colorectal cancer [100]. Invasion of epithelial cells by *P. gingivalis* causes suppression of the apoptotic pathways JAK1, STAT3 and Akt [110, 111] and stimulates cell proliferation [112].

The role of other genera overrepresented in our CRC cohort is less clear. In our study, *Clostridium, Butyricimonas, Peptostreptococcus*, and *Ruminococcus* were significantly overrepresented in CRC samples. Conversely, *Faecalibacterium prausnitzii*, an anti-inflammatory commensal bacterium able to block NF-κB and IL-8 secretion [113, 114] was significantly overrepresented in healthy individuals. Finally, we observed a non-significant overrepresentation of the beneficial bacteria *Lactobacillus* and *Bifidobacterium* in healthy individuals.

Predictive functional analysis of sequencing data showed a higher significant relative abundance of genes responsible for cellular processes including bacterial chemotaxis, bacterial motility proteins, and flagellar assembly in CRC samples. Flagellin is the primary component of bacterial flagella. This protein has the potential to bind to Toll-like receptor 5 (TLR5) activating the transcription nuclear factor-κβ (NF-κβ) signaling-pathway with inflammatory and anti-apoptotic outcomes [115, 116]. Additionally, we found that acetaldehyde dehydrogenase and acetyl-CoA synthetase involved in glycolysis/gluconeogenesis were overrepresented in CRC samples. It is well known that acetaldehyde is highly toxic and is recognized as a carcinogenic molecule to humans [117]. Moreover, acetaldehyde is considered a CRC biomarker and plays a crucial role in cancer initiation and progression [118]. Finally, relative abundance of genes of the pentose phosphate pathway was significantly higher in CRC samples. This pathway plays a critical role in cancer cells by generating high levels of NADPH, which may be used in the synthesis of nucleic acids and is also required for both fatty acids synthesis and cell survival under stress conditions [119, 120].

This is the first study conducted on the CRC-associated gut microbiome in the Moroccan population. Studies have shown that populations from different geographic locations may have different healthy and disease-associated microbiota composition [20, 39, 121, 122], making this study of particular relevance. The low number of samples limited the power of this study; however, the study had the advantage of giving a first insight into the CRC gut microbiota composition of the Moroccan population. Likewise, the use of self-reported dietary information could have resulted in less accurate data. Although self-reported data are one the most used methods for data collection in health research, it could introduce biases and impact data reliability in analysis and potentially, the validity of the conclusions. Future, large-scale gut microbiome studies will confirm data from our pilot study in order to better understand the role of nutrition and other environmental factors on cancer etiology in the Moroccan population.

## Electronic supplementary material

Below is the link to the electronic supplementary material.

**Supplementary Table 1**. Predicted functional metagenome significantly over- or underrepresented in the colorectal cancer and control samples (DOCX 46 KB)
