# Supplementary material for: Gut microbiome of Moroccan colorectal cancer patients
Source: Med Microbiol Immunol. 2018 Apr 23;207(3):211–25. doi: 10.1007/s00430-018-0542-5 (PMC6096775; doi:10.1007/s00430-018-0542-5)
Supplement: Supplementary file 1 — Supplementary Table 1. Predicted functional metagenome significantly over- or underrepresented in the colorectal cancer and control samples (DOCX 46 KB) [file 430_2018_542_MOESM1_ESM.docx]

**Supplementary Tables**

**Supplementary Table 1**. Predicted functional metagenome significantly over- or underrepresented in the colorectal cancer and control samples

|  | **Metabolic Pathways** | | | ***P*-values** | **Control** | **CRC** | **Ratio** | **Fold change** |
| --- | --- | --- | --- | --- | --- | --- | --- | --- |
| **Over represented in the Control Samples** | **Environmental Information Processing** | Signaling Molecules and Interaction | Cellular antigens | 0.0089 | 0.380891634 | 0.351488864 | 1.083652068 | 0.080336881 |
|  | **Genetic Information Processing** | Folding, Sorting and Degradation | Chaperones and folding catalysts | 0.0178 | 0.318472412 | 0.179577949 | 1.77344943 | 0.572926481 |
|  |  |  | Protein processing in endoplasmicreticulum | 0.0337 | 0.027873956 | 0.012516214 | 2.227027771 | 0.800667858 |
|  |  |  | RNA degradation | 0.0247 | 0.479447102 | 0.372072405 | 1.28858549 | 0.253545097 |
|  | **Human Diseases** | Immune System Diseases | Primary immunodeficiency | 0.0035 | 0.233126551 | 0.211597596 | 1.101744799 | 0.096895104 |
|  |  | Infectious Diseases | Vibrio cholerae pathogenic cyclE | 0.0455 | 0.049960891 | 0.039012135 | 1.28065002 | 0.247367778 |
|  |  | Metabolic Diseases | Type II diabetes mellitus | 0.0151 | 0.050319066 | 0.021287071 | 2.363832241 | 0.860284133 |
|  | **Metabolism** | Amino Acid Metabolism | Glycine, serine and threonine metabolism | 0.0178 | 0.431914664 | 0.346271232 | 1.247330488 | 0.221005658 |
|  |  | Carbohydrate Metabolism | Citrate cycle (TCA cycle) | 0.0392 | 0.834017366 | 0.589920312 | 1.413779706 | 0.34626676 |
|  |  | Energy Metabolism | Carbon fixation in photosynthetic organisms | 0.0455 | 0.262602879 | 0.22124084 | 1.186954811 | 0.171391045 |
|  |  |  | Oxidative phosphorylation | 0.0178 | 0.094217741 | 0.053315501 | 1.767173515 | 0.569381386 |
|  |  | Glycan Biosynthesis and Metabolism | Glycosyltransferases | 0.0051 | 0.595088933 | 0.417307404 | 1.426020546 | 0.35488773 |
|  |  |  | Lipopolysaccharide biosynthesis proteins | 0.0089 | 0.081958065 | 0.079595356 | 1.029684008 | 0.029251967 |
|  |  |  | Lipopolysaccharide biosynthesis | 0.0151 | 0.052731666 | 0.046072693 | 1.144531893 | 0.134995726 |
|  |  | Lipid Metabolism | Arachidonic acid metabolism | 0.021 | 0.467642947 | 0.292524697 | 1.598644324 | 0.469155972 |
|  |  | Metabolism of Cofactors and Vitamins | Folate biosynthesis | 0.0023 | 0.305825828 | 0.24993716 | 1.223610879 | 0.201806224 |
|  |  |  | Retinol metabolism | 0.0247 | 1.201727384 | 1.016907191 | 1.181747355 | 0.166994153 |
|  |  |  | Riboflavin metabolism | 0.0151 | 0.882945347 | 0.837410047 | 1.054376348 | 0.052949453 |
|  |  |  | Ubiquinone and other terpenoid-quinone biosynthesi | 0.0006 | 1.267091515 | 1.121525753 | 1.129792616 | 0.122034091 |
|  |  | Metabolism of Other Amino Acids | beta-Alanine metabolism | 0.0023 | 0.066495378 | 0.037029338 | 1.7957485 | 0.585421927 |
|  |  |  | Glutathione metabolism | 0.0062 | 0.51737744 | 0.46835224 | 1.104675917 | 0.099552004 |
|  |  |  | Selenocompound metabolism | 0.0005 | 0.045262018 | 0.028219834 | 1.603907981 | 0.472443139 |
|  |  | Metabolism of Terpenoids and Polyketides | Biosynthesis of siderophore group nonribosomal | 0.0042 | 3.76624007 | 3.613747869 | 1.042197797 | 0.04133175 |
|  |  |  | Geraniol degradation | 0.0127 | 0.145514232 | 0.111782102 | 1.301766825 | 0.263722438 |
|  |  |  | Zeatin biosynthesis | 0.0392 | 0.057973499 | 0.02637142 | 2.198345762 | 0.787705151 |
|  |  | Xenobiotics Biodegradation and Metabolism | Aminobenzoate degradation | 0.0337 | 0.088159984 | 0.06913462 | 1.275193011 | 0.243097548 |
|  |  |  | Toluene degradation | 0.0289 | 0.173942046 | 0.145829559 | 1.192776327 | 0.176283638 |
|  | **Organismal Systems** | Digestive System | Carbohydrate digestion and absorption | 0.0015 | 0.049508095 | 0.041800127 | 1.18440058 | 0.169236807 |
|  |  |  | Protein digestion and absorption | 0.0289 | 0.78021935 | 0.667855433 | 1.168245869 | 0.155503366 |
|  |  | Immune System | NOD-like receptor signaling pathway | 0.0337 | 0.07388279 | 0.056170026 | 1.31534192 | 0.274096647 |
|  | **Unclassified** | Cellular Processes and Signaling | Cell division | 0.0455 | 0.088239496 | 0.077870564 | 1.133155983 | 0.125006645 |
|  |  | Poorly Characterized | Membrane and intracellular structural molecules | 0.0051 | 0.692280067 | 0.647400126 | 1.069323343 | 0.067026059 |
|  |  |  | General function prediction only | 0.0247 | 0.094047819 | 0.077889448 | 1.207452628 | 0.188512874 |
| **Over represented in the Colorectal Cancer Samples** | **Cellular Processes** | Cell Motility | Bacterial chemotaxis | 0.0023 | 0.044838946 | 0.092974515 | 0.482271358 | -0.72924834 |
|  |  |  | Bacterial motility proteins | 0.0051 | 0.257380367 | 0.532358596 | 0.483471798 | -0.726762295 |
|  |  |  | Flagellar assembly | 0.0127 | 0.750349426 | 0.935771051 | 0.801851506 | -0.220831843 |
|  | **Environmental Information Processing** | Membrane Transport | ABC transporters | 0.0392 | 0.514149163 | 1.123338157 | 0.457697586 | -0.781546605 |
|  |  |  | Transporters | 0.0289 | 0.58871833 | 0.693445591 | 0.848975518 | -0.163724929 |
|  |  | Signal Transduction | Two-component system | 0.0178 | 0.253043879 | 0.290084737 | 0.872310214 | -0.136610168 |
|  | **Genetic Information Processing** | Transcription | Transcription factors | 0.0337 | 0.790743143 | 0.855778696 | 0.924004239 | -0.07903862 |
|  |  | Translation | RNA transport | 0.0247 | 1.105636286 | 1.237504885 | 0.893439937 | -0.112676169 |
|  | **Human Diseases** | Immune System Diseases | Systemic lupus erythematosus | 0.0173 | 0.216704024 | 0.445868464 | 0.486026804 | -0.721491505 |
|  |  | Infectious Diseases | Amoebiasis | 0.0127 | 0.006158819 | 0.009157755 | 0.672524929 | -0.396716099 |
|  | **Metabolism** | Amino Acid Metabolism | Histidine metabolism | 0.0247 | 0.03890058 | 0.044976994 | 0.864899504 | -0.145141959 |
|  |  |  | Valine, leucine and isoleucine biosynthesis | 0.0062 | 0.401077223 | 0.673519469 | 0.595494623 | -0.518362919 |
|  |  | Biosynthesis of Other Secondary Metabolites | Butirosin and neomycin biosynthesis | 0.0455 | 1.93206E-05 | 0.000802341 | 0.024080305 | -3.72636098 |
|  |  |  | Flavone and flavonol biosynthesis | 0.021 | 1.226106042 | 1.603984836 | 0.76441249 | -0.268647728 |
|  |  |  | Flavonoid biosynthesis | 0.0246 | 0.259750116 | 0.280935106 | 0.924591159 | -0.078403629 |
|  |  | Carbohydrate Metabolism | C5-Branched dibasic acid metabolism | 0.0089 | 0.004415257 | 0.005661401 | 0.779887607 | -0.248605463 |
|  |  |  | Pentose phosphate pathway | 0.0089 | 0.015121604 | 0.027765526 | 0.544617975 | -0.607670694 |
|  |  |  | Propanoate metabolism | 0.0392 | 0.133694224 | 0.184031887 | 0.726473148 | -0.319553758 |
|  |  | Energy Metabolism | Methane metabolism | 0.0089 | 0.004039 | 0.004991247 | 0.809216679 | -0.211688563 |
|  |  | Enzyme Families | Protein kinases | 0.0392 | 0.108525155 | 0.123325801 | 0.87998743 | -0.127847656 |
|  |  | Lipid Metabolism | Fatty acid biosynthesis | 0.0247 | 0.573585579 | 0.654342111 | 0.876583624 | -0.131723173 |
|  |  |  | Fatty acid metabolism | 0.0455 | 0.457897181 | 0.488980136 | 0.936433093 | -0.065677203 |
|  |  |  | Linoleic acid metabolism | 0.0455 | 0.006032492 | 0.005970788 | 1.010334259 | 0.010281225 |
|  |  | Metabolism of Cofactors and Vitamins | Porphyrin and chlorophyll metabolism | 0.0035 | 4.968552027 | 6.159544809 | 0.806642728 | -0.214874425 |
|  |  | Metabolism of Terpenoids and Polyketides | Biosynthesis of ansamycins | 0.0455 | 1.357783491 | 1.608924043 | 0.843907764 | -0.169712075 |
|  |  |  | Carotenoid biosynthesis | 0.0392 | 0.204748525 | 0.249226164 | 0.821537038 | -0.196578257 |
|  |  | Xenobiotics Biodegradation and Metabolism | Atrazine degradation | 0.021 | 2.535204546 | 3.191624003 | 0.79433058 | -0.230255557 |
|  |  |  | Benzoate degradation | 0.0337 | 0.483420957 | 0.548723588 | 0.880991756 | -0.126707011 |
|  |  |  | Chloroalkane and chloroalkene degradation | 0.021 | 0.264418027 | 0.308592819 | 0.856850875 | -0.154491384 |
|  |  |  | Dioxin degradation | 0.0392 | 0.002929303 | 0.003690023 | 0.79384422 | -0.230868033 |
|  |  |  | Nitrotoluene degradation | 0.0015 | 0.035920003 | 0.049000337 | 0.733056237 | -0.310532858 |
|  |  |  | Xylene degradation | 0.0392 | 0.031298164 | 0.044356903 | 0.70559849 | -0.348708915 |
|  | **Organismal Systems** | Environmental Adaptation | Circadian rhythm – plant | 0.0493 | 0.019434066 | 0.021281143 | 0.913205922 | -0.09079388 |
|  | **Unclassified** | Cellular Processes and Signaling | Electron transfer carriers | 0.0392 | 0.063639146 | 0.072291285 | 0.8803156 | -0.127474799 |
|  |  |  | Germination | 0.0127 | 0.240500587 | 0.272165127 | 0.883656879 | -0.123686438 |
|  |  |  | Other transporters | 0.0178 | 0.055554953 | 0.061550147 | 0.902596603 | -0.102479556 |
|  |  |  | Sporulation | 0.0151 | 0.09734347 | 0.113855365 | 0.854974819 | -0.156683262 |
|  |  | Genetic Information Processing | Transcription related proteins | 0.0247 | 3.12102E-05 | 0.000355059 | 0.087901534 | -2.431538022 |
